# Supplementary figures and images for: Electrical synapse structure requires distinct isoforms of a postsynaptic scaffold
Source: PLoS Genet. 2023 Nov 27;19(11):e1011045. doi: 10.1371/journal.pgen.1011045 (PMC10703405; doi:10.1371/journal.pgen.1011045)

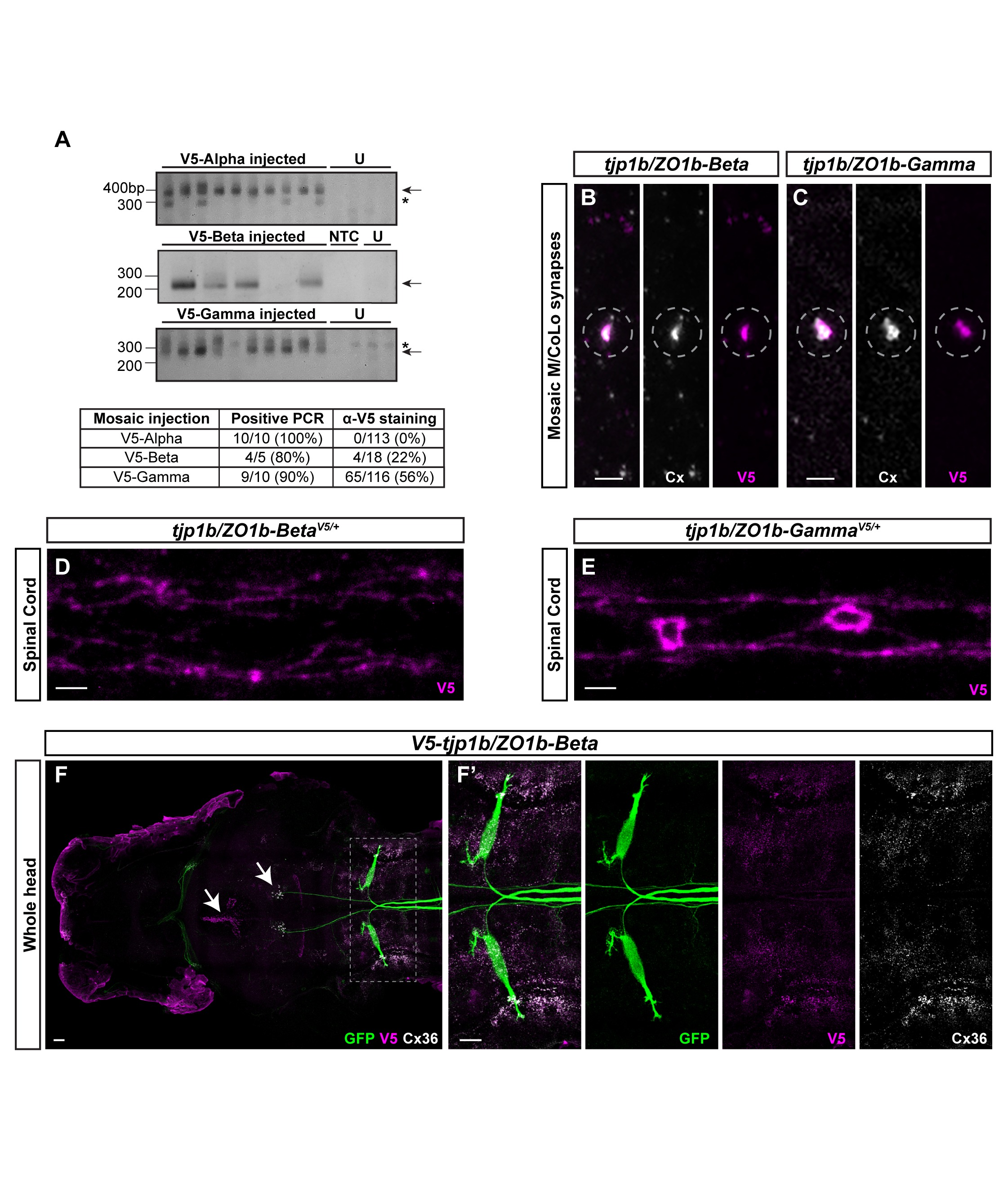

Supplement: S1 Fig — A. Detection of V5-N-terminal tag integration by CRISPR/Cas9 mediated HDR using a short, single-stranded nucleotide repair oligo. Genomic DNA prepared from individual injected 5 dpf zebrafish was analyzed for successful integration by PCR using a forward primer against the V5 tag and a reverse primer outside the modified region. Products for V5-Alpha (top), V5-Beta (middle) and V5-Gamma (bottom) were resolved by agarose gel electrophoresis (indicated by black arrows). Non-specific products are indicated with an asterisk (*). U = uninjected siblings, NTC = no template control. The table shows the percentage of siblings positive for integration and the percentage of siblings positive for V5 immunostain at any body location in the animal, indicating mosaic expression of V5-tagged ZO1b isoforms. B,C. Confocal images of the sites of contact of Mauthner/CoLo processes in the spinal cord of 5 dpf zebrafish larvae mosaically expressing V5-tjp1b/ZO1b-Beta (B) and V5-tjp1b/ZO1b-Gamma (C). V5-tjp1b/ZO1b-Beta animals are stained with anti-Cx35.5 (white), and anti-V5 (magenta). V5-tjp1b/ZO1b-Gamma animals are stained with anti-Cx36 (white), and anti-V5 (magenta). Anterior up. Scale bars = 2 μm. Images are maximum-intensity projections of ~3–4 μm and the dashed circle denotes the M/CoLo site of contact. Neighboring panels show individual channels. D,E. Confocal images of spinal cord floor plate collected from heterozygous V5-tjp1b/ZO1b-Beta (D) and heterozygous V5-tjp1b/ZO1b-Gamma (E) animals. Animals are stained with anti-V5 (magenta). Scale bars = 2 μm. Images are maximum-intensity projections of ~4 μm. Anterior left. F. Confocal tile scan of zebrafish brain from 5 dpf zf206Et zebrafish larvae from V5-tjp1b/ZO1b-Beta animals. Images are maximum intensity projections of ~42 μm. Animals are stained with anti-GFP (green), anti-V5 (magenta), and anti-Cx36 (white). Scale bars = 20 μm. Boxed region denotes stereotyped location of electrical synapses where V5-ZO1b-Beta and Cx36 overla [file pgen.1011045.s001.tif]

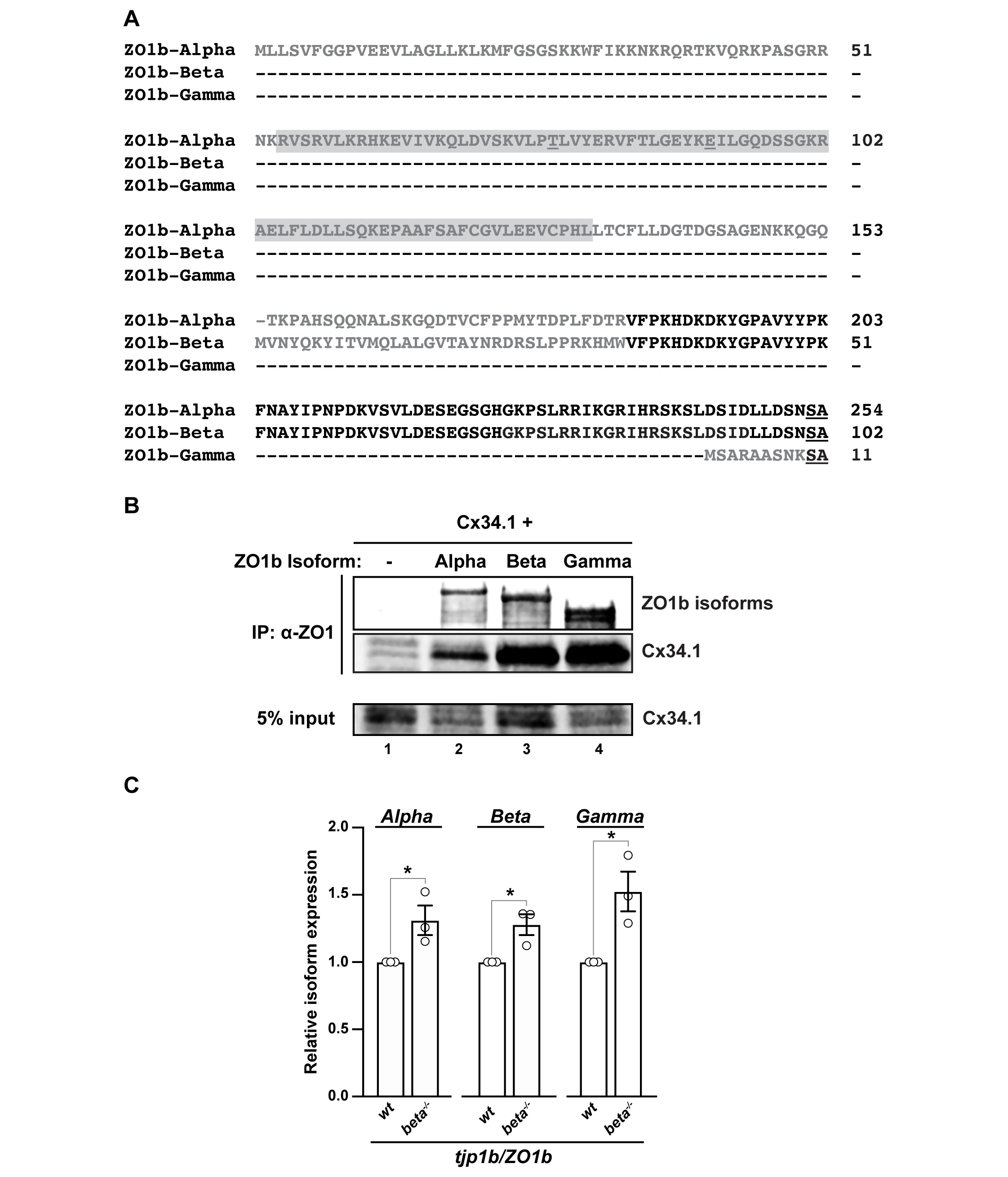

Supplement: S2 Fig — A. Amino acid sequence alignment of predicted unique N-termini from ZO1b isoforms. Amino acids in grey are unique to each isoform, and amino acids in black are shared between isoforms. Amino acids in black underline indicate the beginning of the sequence common to all isoforms (encoded by exons 5–31). The grey box outlining amino acids in ZO1b-Alpha indicates the CARD domain. Amino acids in grey underline in the CARD domain indicate residues that differ between the published predicted sequence and the cloned sequence tested in (B). B. ZO1b isoform interaction with Cx34.1. HEK293T/17 cells were transfected with plasmids to express Cx34.1 and empty vector (lane 1), ZO1b-Alpha (lane 2), ZO1b-Beta (lane 3), or ZO1b-Gamma (lane 4). Lysates were immunoprecipitated with anti-ZO1 antibody and analyzed by immunoblot for the presence of ZO1b isoform (upper) using anti-ZO1 antibody and Cx34.1 protein using Cx34.1-specific antibody (middle). Total extracts (bottom, 5% input) were blotted for Cx34.1 to demonstrate equivalent expression. Results are representative of three independent experiments. C. qPCR analysis of ZO1b isoform mRNA levels in wt and tjp1b/ZO1b-beta-/- mutants. Relative expression of each ZO1b isoform was determined after normalizing to a reference gene using the 2-ΔΔCT (Livak) method. The height of the bar represents the relative fold-expression in tjp1b/ZO1b-beta-/- mutants compared to wt for each tjp1b/ZO1b isoform, as labeled. In wt, n = 3 for each tjp1b/ZO1b isoform tested. In tjp1b/ZO1b-beta-/- mutants, n = 3 for each tjp1b/ZO1b isoform tested. Circles represent results of three independent experiments. Mean ± SEM is shown. Transcriptional upregulation of all isoforms is observed. In Alpha, * indicates p = 0.0472 by unpaired t-test. In Beta, * indicates p = 0.0234 by unpaired t-test. In Gamma, * indicates p = 0.0232 by unpaired t-test. Note that the tjp1b/ZO1b-Beta CRISPR transcript does not undergo nonsense mediated decay. (TIF) [file pgen.1011045.s002.tif]

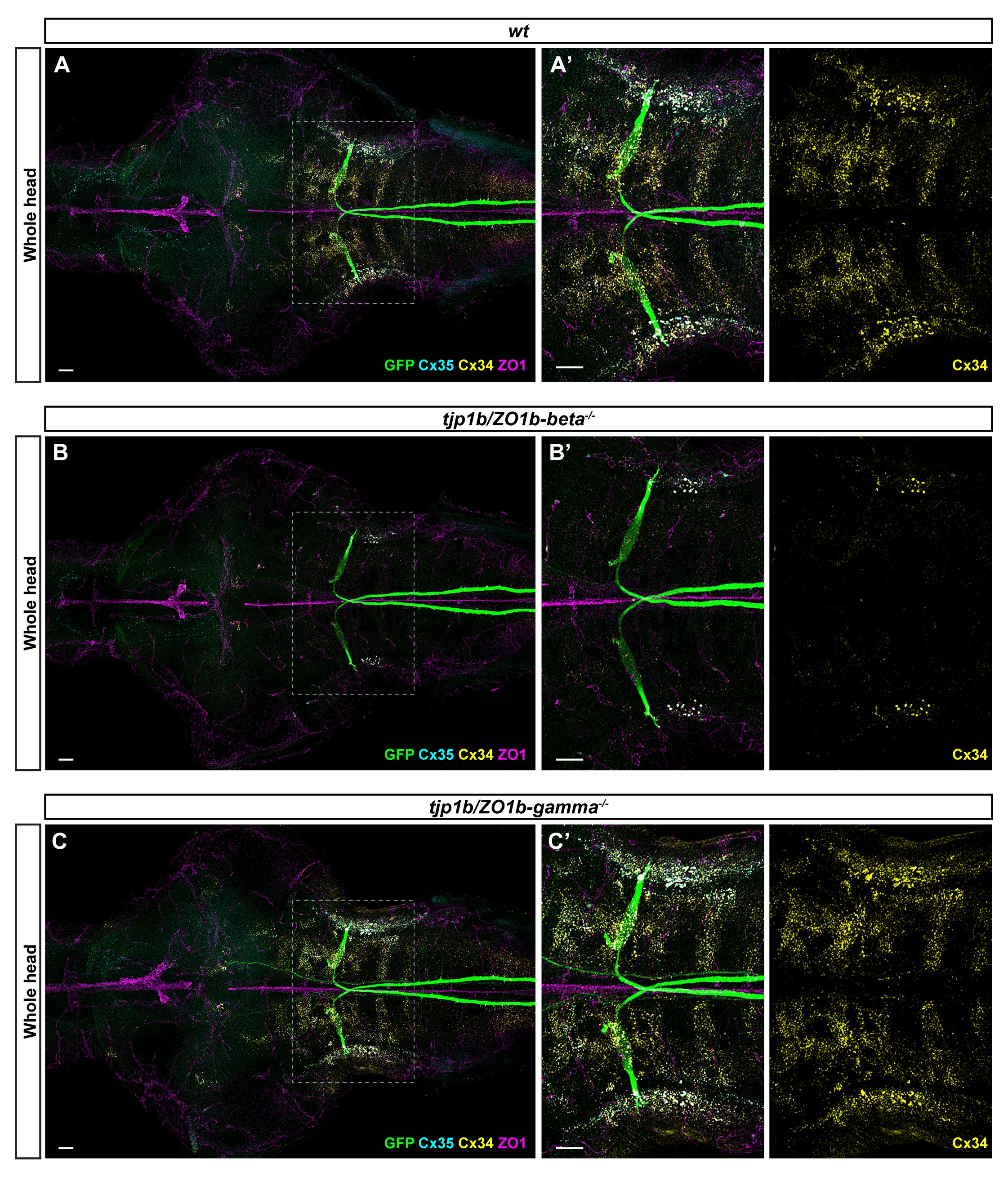

Supplement: S3 Fig — A-C. Confocal tile scan of zebrafish brain from 5 dpf zf206Et zebrafish larvae from the indicated genotypes. Images are maximum intensity projections of ~46 μm. Animals are stained with anti-GFP (green), anti-Cx35.5 (cyan), anti-Cx34.1 (yellow), and ZO1 (magenta). Scale bars = 20 μm. Boxed region denotes stereotyped location of electrical synapses where ZO1b-Beta and Connexins overlap, and the region is enlarged in A’-C’ with the neighboring panel showing the Cx34.1 channel. Anterior left. (TIF) [file pgen.1011045.s003.tif]

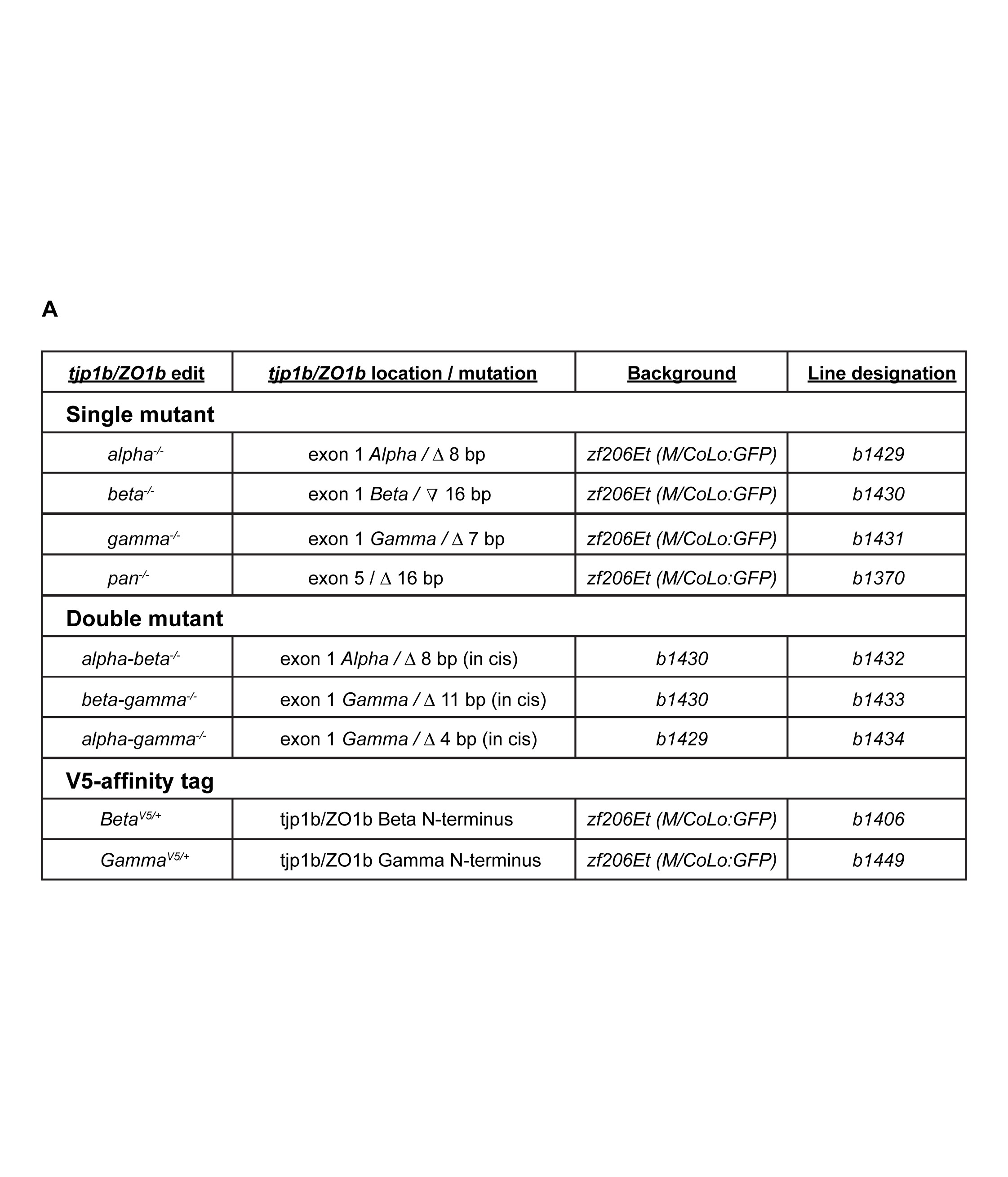

Supplement: S4 Fig — A. A list of single mutant, double mutant, and V5-affinity tagged animals. Columns detail the tjp1b/ZO1b isoform(s) targeted for edit, the exon location and mutation recovered, the background in which the animal was generated, and the line designation assigned. (TIF) [file pgen.1011045.s004.tif]
