# Supplementary material for: Electrical synapse structure requires distinct isoforms of a postsynaptic scaffold
Source: PLoS Genet. 2023 Nov 27;19(11):e1011045. doi: 10.1371/journal.pgen.1011045 (PMC10703405; doi:10.1371/journal.pgen.1011045)
Supplement: Attachment — Submitted filename: Micheletal_PLOSGenetics_response_final.pdf [file pgen.1011045.s006.pdf]

Dear Mary and Gregory,

Thank you very much for evaluating our Research Article entitled 'Electrical synapse formation is differentially regulated by distinct isoforms of a postsynaptic scaffold' for PLOS Genetics. We have modified the manuscript according to the three independent reviewer recommendations. Below is a summary of the changes and a point-by-point response to each reviewer comment. Our responses to Reviewers (below) and the major changes to the manuscript appear in blue.

Title: Upon suggestion by the reviewer, the new title of the paper is "Electrical synapse structure requires distinct isoforms of a postsynaptic scaffold".

Authors: We have added two authors that contributed to the revised manuscript.

Figures: In response to the Reviewers, we reconfigured the figures and moved much of the Supplemental data into the main figures and included the requested new quantification data. This led to the major changes to the figures, listed below.

1. **New Figure 1:** Original figure 1 was modified by removing panels D-O. The Mauthner circuit model in panel A was clarified by reconfiguring the cartoon for easier interpretation. Single panel C was clarified as requested by breaking the mRNA and protein structures into two separate panels: now new Figure 1 C, D.
2. **New Figure 2:** Data from original Supplemental figure 1 (panels A, B; now new Figure 2A,B) and original figure 1 (panels D-O; now new Figure 2C-N) were combined to create New Figure 2.
3. **New Figure 3:** Original figure 2 was modified by removing panel K and is now relabeled as New Figure 3. Panels A, C, E, G, H, I, and J were updated to reflect the new quantitation data requested by reviewers.
4. **New Figure 4:** This figure accommodates new quantification data requested from the reviewers. New Figure 4A contains new, Reviewer requested, quantification data for CE Synapses for Cx35.5, Cx34.1 and ZO1 for single isoform mutants. New Figure 4B contains new quantification data for M/CoLo synapses for Cx35.5, Cx34.1, and ZO1 for single isoform mutants. Data from original figure 2 (panel K) was removed, re-quantitated alongside Cx35.5 and ZO1 channels, and presented in new figure 4B.
5. **New Figure 5:** Data from original Figure 3A-H (now new Figure 5A-H) and original Figure 3I (now presented in new Figure 5J) were combined with new, Reviewer requested, quantitation data to create New Figure 5. New Figure 5 contains new quantification data for Cx35.5, Cx34.1 and ZO1 for double isoform mutants for CE synapses (I) and M/CoLo synapses (J).
6. **New Figure 6:** New Figure 6 presents a Reviewer requested model to summarize the contribution of each *tjp1b/ZO1b* isoform to electrical synapse formation in the Mauthner circuit.
7. **New Supplemental Figure 1:** Original Supplemental figure 1 was modified by moving panels A, B into the main figure, as requested. Original supplemental figure 1 panels C-H were relabeled (new Fig. S1A-F) .

8. **New Supplemental Figure 2:** All data from original Supplemental figure 2 remains the same with the exception that panel C was re-formatted into a bar graph for easier interpretation. This was left as a supplement as it does not provide data that contributes to the main findings of the work.
9. **New Supplemental Figure 3:** New data requested by the reviewers to demonstrate gross synaptic Connexin localization in the brains of mutant fish.
10. **New Supplemental Figure 4:** As requested by the reviewers, a summary of all mutant and transgenic fish used in this study are presented.

Statistics: Reviewers pointed out that ANOVA is the appropriate statistical test to compare multiple categories. We re-analyzed the data and updated the new figures (new Fig. 5A,B and new Fig. 6I,J), their respective figure legends and Results section to reflect the new statistical significance and test performed. A new subsection for statistical analysis in the Materials and Methods is also added. Raw data values, computed data as shown in figures, and information on statistics, is included in the "Source Data" table.

New quantitation data: The results of the new quantitation data are added to the figures (detailed above), the results section, and the discussion section.

Discussion: Points of clarification identified by the reviewers were added as detailed in the point-by-point response below.

Point-by-point response to reviewer comments (**our responses are in blue**):

We appreciate the extremely constructive criticism from all three Reviewers. In particular we would like to highlight the new quantitative analysis provided for all single isoform and double isoform mutants – this reviewer-requested data was very enlightening and helped us streamline and clarify the manuscript. We appreciate the opportunity to revise and hope the Reviewers agree that this has improved the presentation and clarity of the work.

Reviewer #1:

Comments to the Authors:
Reviewer #1: In a previous study, this research group identified the function of Zonula Occludens 1 (ZO1) as a critical scaffolding protein for the recruitment &/or stabilization of Connexin proteins to form functional neuronal gap junctions. In this follow-up study, they use the same larval zebrafish model of electrical synapses in Mauthner Cells to evaluate how three distinct isoforms of ZO1b (-Alpha, -Beta, and -Gamma) influence the localization and accumulation of gap junction proteins Cx34.1 and Cx35.5. They observed expression and protein localization of both -Beta and -Gamma isoforms at Club Ending and Mauthner/CoLo electrical synapses. Using single, double, and triple knockout of ZO1b isoforms, the authors then evaluated the relative contribution of each isoform to the localization of electric synaptic gap junction components. These

experiments revealed a predominant role for ZO1b -Beta in localizing Cx34 and Cx35 and support that distinct isoforms of ZO1 play unique roles in the assembly of gap junction components.

Overall, the experiments are rigorous and provide important anatomical evidence that diversity of ZO1 isoforms regulates the assembly of gap junctions at electrical synapses. Minor revisions are recommended regarding the interpretation of the knockout results. While I agree with the conclusion that ZO1b-beta plays a prominent role in electrical synapse formation, the anatomical data alone do not definitively demonstrate that the beta isoform is necessary and sufficient to form electrical synapse. Fig 3 E-H shows smaller, but still formed, electrical synapses in fish lacking the Beta isoform compared to the ZO1b-pan knockout in Fig 2C. Further, the presence of the Beta isoform (Fig 3C) alone leads to the formation of abnormally large synapses, which could also alter synaptic function.

Point by point comments and recommendations are below:
- It is unclear why two major figures discussed in the results are presented as supporting data. The manuscript would be strengthened by making the supporting figures "Fig 1 and Fig 2", especially since they make up a considerable part of the results section.

Thank you for this suggestion. We have reconfigured the presentation of the data, moving much of the supplemental data to the main figures.

- ZO1b-beta -/- (Fig 3G and associated results): While it was shown that the Alpha and Gamma isoforms are upregulated in this mutant, is notable that the accumulation of ZO1 protein appears (at least qualitatively) to be comparably as diminished in accumulation at the postsynapse as the pre- and postsynaptic Connexin proteins. Given that all three isoforms of ZO1b can interact with Cx34.1 (Fig S2B), could the observed difference be that more Beta isoform is able to accumulate &/or be retained at the electrical postsynapse (i.e. it is not that Beta is a better scaffold for Cx34, it's just that there's more scaffold for Cx34)?

Thank you for this comment, and we agree with this point. We mentioned this idea previously in the Discussion, but we now add language to clarify the point (line 294-295). In addition, we note that in response to the Reviewers we added quantitation of Cx35.5, Cx34.1, and ZO1 for all isoform mutants – one striking finding is that the beta-/- and gamma-/- mutants result in the same decrease in ZO1 antibody staining at CE and M/CoLo synapses, yet only beta-/- mutants results in a decrease in synaptic Cx35.5 and Cx34.1 localization (New Fig.4). The similar decrease in ZO1 staining in both beta-/- and gamma-/- mutants suggest each isoform is present at the synapse at similar levels, though with distinct functions. However, we do not push this point in the paper as the ZO1 antibody is imperfect to assess beta/gamma levels. Indeed, we have previously shown this antibody binds to not only ZO1b but other proteins in zebrafish brain, likely including ZO1a and the related scaffolds ZO2a and ZO2b (Marsh et al, Current Biology;

Lasseigne et al, eLife). Emerging evidence from our lab (not yet published) has found that ZO1a and both ZO2a and ZO2b also co-localize to these synapses. Given these challenges, we use the Discussion to address both the possibility of different scaffold isoform levels as well as different isoform functions (lines 292-315).

- It is intriguing that loss of Alpha or Gamma isoform alone does not contribute to changes in Cx34 accumulation (Fig 2 E,I), but that loss of both leads to a significant increase in Cx34. It is possible that the Gamma isoform may be playing an important role in regulating postsynaptic density size, and Alpha may be able to compensate for the loss Gamma (which would be unusual given that Alpha doesn't normally localize to these synapses)?

Thank you for this idea. Gamma may indeed be playing an important role in regulating postsynaptic electrical density size at M/CoLo synapses. Yet, the role is ultimately obscured in our current analysis by, we think, a combination of cell biological differences between CE and M/CoLo synapses and by potential compensation in the mutants. Our original submission contained quantitation for only the M/CoLo synapses in the double mutants and, as noted in the question, the alpha-gamma-/- double mutants show increased Connexin localization (Fig.5J). However, in new reviewer-requested quantitation data for CE synapses, the alpha-gamma-/- double mutants show a small decrease in synaptic Connexin localization (Fig.5I). Why are the effects opposite for the two different synapse types?

We do not have a definitive answer currently. Yet, emerging evidence is beginning to reveal that these two synapses, despite being connected by Mauthner, are diverse in their mechanisms of assembly. CE synapses are made between auditory afferent axons from cranial nerve VIII and the lateral Mauthner dendrite, while M/CoLo synapses are made between the Mauthner axon and CoLo neurites. We have shown previously that ZO1b and Cx34.1 are exclusively postsynaptic at each of these synapses – cell biologically, that means the Mauthner dendrite contributes ZO1b/Cx34.1 to CE synapses but not to M/CoLo synapses (Lasseigne et al, eLife, 2021; Miller et al, eLife, 2017). Instead, the Mauthner axon provides Cx35.5 at M/CoLo synapses, while the CoLo contributes ZO1b/Cx34.1 to M/CoLo synapses (Lasseigne et al, eLife, 2021; Miller et al, eLife, 2017). These distinct cellular contributions lead us to hypothesize that there could be compartmental-specific differences in protein trafficking of the different ZO1b isoforms (and other asymmetric proteins) that may explain these distinct effects on CE and M/CoLo synapses in the alpha-gamma-/- double mutants. Yet definitive results are ultimately challenging given they require mosaic analysis, achieved by cellular transplant between animals, combined with fluorescently tagged Connexins (as we did in Martin et al, Current Biology, 2023).

Alternative to this 'trafficking hypothesis', the observed differences may be due to cellular differences emerging from compensation (Fig. S2C), perhaps with upregulated expression occurring at different levels in each of the cells contributing to the different synapses. Given these complications, we do not make strong claims from our data as to

the function of the Gamma isoform. We did add a brief discussion of the idea of compartment specific differences to the result sections (lines 268-270). We also have a section of the Discussion addressing *potential* Gamma isoform functions (lines 302-315) – but we believe that experimental evidence will take a combination of complex genetics (as noted above) combined with cell biological, electrophysiological, and biochemical analysis to reveal the role of the Gamma isoform.

- In the interest of open and transparent science, data that is discussed and is not published needs to be shown as a supporting figure (referring to loss of gross synaptic Connexin in the brain in the Beta -/- (but not Gamma -/-) mutants). Alternatively, the authors can remove reference to this observation from the discussion.

Thank you for this suggestion. We have added this data and present it in the new Supplemental Figure 3.

- A statistical analysis subsection needs to be added to the Materials and Methods section.

Thank you for this suggestion. We have added a statistical analysis subsection to the Materials and Methods section. Additionally, all raw and processed data, and statistical tests, can be found in the 'Source Data' table.

Reviewer #2:

This is a beautifully written manuscript supporting the novel claim that distinct protein isoforms of a single gene (ZO1b) make unique contributions to electrical synapse density (ESD). The significance of this work is clear: we know very little about the complex proteome of the ESD; this work demonstrates that the complexity of the ESD is not limited to gene identity, but is further complicated by isoform-specific roles in electrical synapse structure and function. The authors generate a variety of novel mutants and genetic tools specific to each of three ZO1b isoforms. They use these tools to demonstrate that two of the three isoforms are present at stereotyped electrical synapses in the zebrafish, and further show that one isoform, in particular, is essential for localizing connexins to the synapse. The conclusions are well supported by the data.

Major comments:
• T-tests are not appropriate when comparing more than two genotypes. Fig. 2K, Fig. 3I, Fig. S2C should use one-way ANOVAs. This can be easily recalculated in PRISM.

Thank you for this correction. We have recalculated the data in question using ANOVA and corrected the figures, figure legends and results sections accordingly.

For previous Figure S2C, we feel the unpaired t-test is the correct test. The data was re-formatted into a bar graph for easier interpretation and is now presented in new Figure S2C.

• It would be helpful to have a Table summarizing all the (amazing) mutant and transgenic lines that were used in this study.

Thank you for this suggestion. We added a summary of the fish lines generated and used in this study, and we present it in the new Supplemental Figure 4.

• An additional Figure/schematic summarizing the synaptic phenotype of the main mutant isoforms would be helpful (WT/alpha; beta; gamma; beta/gamma/ZO1b-pan) mutants).

Thank you for this suggestion. We added a model summarizing the contribution of each ZO1b isoform to electrical synapse formation in the Mauthner circuit, and we present it in the new Figure 6.

• As we only see a single representative image, please quantify the Cx34 label at CE synapses in addition to the M/CoLo synapses in Figures 2/3.

Thank you for this suggestion. Based on your suggestion and other reviewer comments, we quantitated Cx35.5, Cx34.1, and ZO1 at both CE and M/CoLo synapses. The new data are presented in new Fig. 4 and new Fig. 5.

Minor comments/suggestions

• Results: We also observed overlapping expression for “all” isoforms in other areas.

Thank you. We have added this correction.

• Results: First section 4th paragraph. It would be helpful to clarify pre/post sites of each synapse here.

Thank you for this suggestion. We have added this clarification in this paragraph (lines 149-150 and 152-153).

• Results. Section 3, 3rd paragraph. While greatly diminished…..mutants retained “residual” Connexin localization…

Thank you. We have added this phrasing to our observations.

• Results. Section 3, 3rd paragraph. First sentence. It is unclear that the %'s are being compared to WT (100%).

Thank you. We have clarified the quantitative data for both the individual isoform mutants and the double mutants. The sections are found on lines 228-239 and 258-272.

• In Fig. 1A, the "Club ending (CE) synapses" label is very close to some M/CoLo synapses; this may be confusing for readers not familiar with the system.

Thank you. We have modified the Mauthner circuit cartoon in new Figure 1A to provide clarification.

• The caption for Fig. 1 is missing a description of panel A (and as such a few panel names are mislabeled).

Thank you. We have corrected the figure legend in new Figure 1.

• Fig. S1 caption, panel A: "Cell[s] are derived…"

Thank you. We have added this correction to the caption (now new Figure 2A).

• In the first paragraph of the into, I think you meant to say "regulate synaptic vesicle release and neurotransmitter [receptor] localization".

Thank you. We have added this correction.

• Based on the data presented in this manuscript, it is difficult to specifically claim that ZO1b is involved in synapse formation vs. maintenance. It is possible that ZO1b is essential for either recruiting Connexins or stabilizing Connexins at the electrical synapse (or both). Are Connexins present at an earlier stage? Perhaps you could replace "formation is differentially regulated by" with "structure requires distinct" in the title?

Yes, we agree that it is difficult to distinguish between formation versus maintenance, and we are currently working to define experimental conditions that will distinguish between these two ideas. Thus, we agree that "Electrical synapse structure requires distinct by distinct isoforms of a postsynaptic scaffold" is a more appropriate title.

• Page and Line numbers would be helpful in any subsequent revisions.

Thank you for the suggestion. We have added page and line numbers to the revised manuscript.

Reviewer #3: Michel 2023 Plos genetics review

This study by Michel et al. provides a clear and concise demonstration of the isoform-specific role of ZO1b in electrical synapse formation in vivo. Fluorescent in situ probes indicate expression of Beta and Gamma ZO1b mRNAs in zebrafish Mauthner and CoLo neurons, but not the Alpha isoform. By inserting a V5 tag into the ZO1b locus at each transcriptional start site, the authors show that protein made from Beta and Gamma colocalizes with pre- and postsynaptic Connexins at electrical synapses in Mauthner and CoLo neurons. All 3 isoforms can interact with Cx34.1 in vitro, but using CRISPR gRNAs to specifically knock out each isoform the authors show that only the Beta isoform is required for normal Cx34/Cx35 expression in vivo. Double-mutant analyses show that the Beta isoform is also sufficient for normal Cx34/Cx35 expression. The design of the study is sound, the manuscript is well-written, and the novel results showing isoform-specific requirements for electrical synapse formation will be of interest to the field. With a few minor revisions the paper's conclusions can be further strengthened.

Key points to address:

Since this is the first study confirming that multiple isoforms of ZO1b are produced in vivo and not just predicted, it would be important to confirm the full sequence of each expressed isoform. Do they match with the predicted sequences and contain the expected protein domains? The authors likely sequenced each isoform for the HEK cell binding assay, so they could simply clarify that the mRNA/protein structures in Fig. 1C were confirmed by sequencing.

Thank you for this suggestion. We modified the text to acknowledge that the full sequence of each isoform used for cell culture experiments was confirmed to contain all the protein-protein interaction domains. Further, ZO1b-Beta and ZO1b-Gamma matched their predicted unique N-termini, respectively. For ZO1b-Alpha, there are two amino acid differences between the predicted and the cloned unique N-terminus. These differences have been noted in in the Materials and Methods section (lines 570-574).

The authors show expression of ZO1b at auditory club ending synapses on Mauthner cell lateral dendrites, but in Fig. 2 and 3 they only quantify expression at the downstream Mauthner/CoLo synapses. Furthermore, they only quantify Cx34.1 intensity. From the images in the figures, it appears that analysis of ZO1 and Cx35 staining would follow the same pattern as for Cx34 at both M/CoLo and club ending

synapses. But including these analyses – from images the authors have already collected – would further strengthen the authors' conclusions, allow for a more complete understanding of how each isoform affects both the pre and postsynaptic side of these electrical synapses, and clarify any potential compensation/adaptation by other ZO proteins.

Thank you for this suggestion. Based on your suggestion and other reviewer comments, we quantitated Cx35.5, Cx34.1, and ZO1 at both CE and M/CoLo synapses. The new data are presented in new Fig. 4 and new Fig. 5.

Along the same lines, the authors mention but say "data not shown" that loss of Beta but not Gamma disrupts Cx expression across the brain. Showing this data would further strengthen the authors' conclusions that Beta is the key isoform required for Cx expression, not just at M/CoLo synapses, but throughout the CNS.

Thank you for this suggestion. We have added this data and present it in the new Supplemental Figure 3.

How do the authors account for the residual Cx labeling in the Alpha-Beta and Beta-Gamma mutants in Fig. 3I? Previously there seemed to be no role for Alpha at these synapses, could ZO1b-Alpha, ZO1a, or ZO2/3 potentially be able to partially compensate for the loss of ZO1b-Beta?

Thank you for this idea and we agree. In the Discussion we have expanded our comments on the complexity of this gene family, and highlight the challenge of compensation / transcriptional adaptation and how this may explain the residual Connexin localization in the *tjp1b/ZO1b-alpha-beta*$^{-/-}$ and *tjp1b/ZO1b-beta-gamma*$^{-/-}$ double mutants. (lines 315-325).

In the Discussion's first paragraph the authors discuss how differential expression of each isoform may occur. Could mRNA or protein degradation of non-Beta forms also explain or contribute to the observed expression differences between isoforms, either in addition to or instead of proposed but unknown genomic regulatory elements upstream of each start site?

Thank you for this idea. Yes, this certainly seems very likely in the mutants and, as noted above, we address this notion in the Discussion (lines 315-325). In wildtype, it could be that mRNA or protein degradation could act as regulatory control mechanism – but we have no evidence to support such. Alternatively, other post-transcriptional mechanisms could certainly be at play to regulate Beta or Gamma isoforms. As we mention in the Discussion, there is little known about transcriptional control of genes involved in electrical synapse formation, and even less known about other forms of 'tuning' mRNA/protein levels to shape the structure and function of the neuronal gap junctions. We wonder whether such tuning control of levels will ultimately (1) set the structure of the gap junction (size, Connexin level) and regulate the function (ionic

communication). We are particularly interested in this concept in terms of electrical synaptic plasticity. But, within the framework of this manuscript, we hope to be circumspect in what we state from the evidence we have on hand.

With the caveat that different labeling methods were used, based on the robust V5 labeling of the Gamma isoform (Fig. 1N,O) at club ending and M/CoLo synapses in comparison to the very weak ZO1 staining of Beta mutants (Fig. 2G,H), it appears that Gamma expression may depend on Beta expression. It's not clear if the construction of the transgenic and mutant lines would allow for this, but analyzing V5-Gamma labeling in Beta mutants vs. siblings could clarify if Gamma expression requires Beta. This could be particularly interesting in light of the fact that Gamma mRNA is upregulated in Beta mutants (Fig S2C), along with the authors' speculation in the Discussion that each isoform may contribute distinct functions to the electrical synapse, analogous to SAP97 and PSD95 at chemical synapses. Minimally, could the authors discuss what they think the role of Gamma may be in normal fish that exhibit robust expression of both Beta and Gamma at Mauthner electrical synapses?

Thank you, we would love to do this experiment. Yet the proposed experiment is impossible with the current animals (which were already a huge challenge to make!). We would need to V5-tag ZO1b-Gamma (CRISPR injection with V5 repair construct) in the various mutant backgrounds, as the fish cannot simply be crossed to achieve the desired genotypes (these modifications must be made 'in cis' on the chromosome of the *tjp1b* locus - e.g. in a beta mutant, introduce the V5 tag into the gamma exon, grow up lines, etc.). As suggested, we have clarified the Discussion to be explicit about the *potential* role Gamma may play in wildtype fish (lines 302-315). And see our comments to Reviewer 1 as well.

Minor points to address:

It would be very helpful to clearly indicate the groups being compared in statistical analysis in Fig 2K. Should include comparison of pan vs beta and beta-gamma mutants.

Thank you for this suggestion. We clarify that the groups are being compared to wildtype in the statistical analysis in new Figure 4 and new Figure 5 by connecting the bars with grey lines.

The methods indicate that multiple M/CoLo synapses were analyzed in each fish and averaged, with n representing the number of fish used. Can the authors clarify that the M/CoLo synapses analyzed were always from the same spinal segments in each fish?

The M/CoLo synapses analyzed were as similar as possible in each fish. The first scan imaged the spinal segments where M/CoLo synapses becomes regularly spaced within the spinal cord, which occurs at approximately somite 10 within the spinal cord. The same number of neighboring, caudal somites were imaged and analyzed in all animals.

Please clarify that fish were imaged and analyzed blind to genotype.

We do not image and analyze blind to genotype. Experiments are done in one of two ways. Genotyped, homozygous parent fish of a particular genotype are crossed on the same day and 5dpf larvae are collected. A sample of siblings are sacrificed to confirm genotypes while the remaining sibling are immunostained. Alternatively, genotyped, heterozygous parent fish of a particular genotype are crossed and 5dpf larvae are collected. After euthanization, the eye of each larvae is removed and genotyped while the remaining fish tissue is fixed. Once the fish have been genotyped, the larvae are sorted into groups and immunostained. Immunostained fish are dissected and mounted onto the same slide as the control to which it is normalized (usually wt unless otherwise indicated). Within each fluorescence experiment for quantification, all animals were stained together using the same antibody mix, processed at the same time, and all confocal settings (laser power, scan speed, gain, offset, objective, and zoom) were identical. Multiple animals per genotype allows us to account for biological variation. To account for technical variation, fluorescence intensity values for each region of each animal were an average across multiple synapses.

Typo: “noteably” should be “notably” on p. 7, 1st paragraph
Thank you. We made the correction.
